# Preparation of an Environmentally Friendly Nano-Insecticide through Encapsulation in Polymeric Liposomes and Its Insecticidal Activities against the Fall Armyworm, *Spodoptera frugiperda*

**DOI:** 10.3390/insects13070625

**Published:** 2022-07-13

**Authors:** Xiuqin Chen, Liangmiao Qiu, Qiquan Liu, Yuxian He

**Affiliations:** Fujian Key Laboratory for Monitoring and Integrated Management of Crop Pests, Institute of Plant Protection, Fujian Academy of Agricultural Sciences, Fuzhou 350013, China; zbchenxiuqin@faas.cn (X.C.); liuqiquan@faas.cn (Q.L.); heyuxian@faas.cn (Y.H.)

**Keywords:** DSPE-PEG_2000_-NH_2_, nanoparticles, sustained release, emamectin benzoate, biodegradable, *Spodoptera frugiperda*

## Abstract

**Simple Summary:**

Pests are an important factor that causes a heavy loss in corn yield and quality. The fall armyworm (FAW), *Spodoptera frugiperda,* is a newly invasive and extremely destructive pest, and it poses a major threat to agricultural production in China. While chemical pesticides are considered effective means for controlling the outbreak of destructive pests, pesticide delivery systems, such as microcapsules or nanoparticles, are an effective way to promote the utilization rate of traditional pesticides and to reduce environmental pollution. Therefore, the aim of this study is to design an environmentally friendly nano-insecticide that can enhance foliar retention and increase insecticidal activity. For this purpose, 1,2-distearoyl-sn-glycero-3-phosphoethanolamine-N-[amino(polyethylene glycol)-2000] (DSPE-PEG_2000_-NH_2_) was chosen to formulate the insecticide nanoparticles. The physicochemical properties were characterized and investigated in indoor and field efficacy trials. The results demonstrate that the nanoparticles hold promise for pest control.

**Abstract:**

The insecticide emamectin benzoate (EB) was formulated with nanoparticles composed of DSPE-PEG_2000_-NH_2_ by the co-solvent method to determine its adverse impacts on the environment and to reinforce its dispersion, adhesion, and biocompatibility. A good encapsulation efficiency (70.5 ± 1.5%) of EB loaded in DSPE-PEG_2000_-NH_2_ polymeric liposomes was confirmed. Dynamic light scattering (DLS), transmission electron microscopy (TEM), and contact angle meter measurements revealed that the DSPE-EB nanoparticles had a regular distribution, spherical shape, and good leaf wettability. The contact angle on corn leaves was 47.26°, and the maximum retention was higher than that of the reference product. DSPE-EB nanoparticles had strong adhesion on maize foliage and a good, sustained release property. The efficacy trial showed that the DSPE-EB nanoparticles had a strong control effect on *S. frugiperda* larvae, with the LC_50_ of 0.046 mg/L against the third-instar *S. furgiperda* larve after 48 h treatment. All these results indicate that DSPE-EB nanoparticles can serve as an insecticide carrier with lower environmental impact, sustained release property, and effective control of pests.

## 1. Introduction

Pesticides are essential for controlling weeds, plant pests, and diseases in order to ensure the food yield [1,2]. However, traditional pesticide formulations use large quantities of organic solvents to improve the water solubility of pesticides, which is considered an unsustainable method [3]. Moreover, the excessive use of traditional pesticides causes serious concerns about insect pest resistance, human health, and environmental safety [4,5]. Due to volatilization, spray drift, runoff, photolysis, and microbial degradation, more than 70–90% of these conventional pesticides can be lost to the surrounding environment during their application [6,7,8]. Therefore, the development of specific environmentally friendly pesticide formulations is an urgent need. Over the past few decades, advanced polymeric materials have gained popularity in the development of sustainable agricultural applications [9,10,11,12]. The encapsulation of polymeric materials can improve the application of hydrophobic pesticides, increasing their solubility in water and the permeability of plant tissues, which in turn enhance the pesticides spreading and wetting properties while reducing the volatilization and degradation of active ingredients and improving the biocompatibility and environmentally friendliness [13,14,15,16,17,18,19,20,21].

Amino-modified DSPE-PEG_2000_ is an amphiphilic polymer with a hydrophilic PEG block and a hydrophobic distearoylphosphatidylethanolamine (DSPE) block. Under aqueous conditions, this polymer self assembles to form a micellar structure [22]. The hydrophilic PEG end forms the corona of the micelle, which shields the pesticide and improves its solubility in water, while the lipidic DSPE end forms the core. DSPE-PEG_2000_-NH_2_ is biodegradable and biocompatible and thus has potential in agriculture. 

*S. frugiperda*, which is native to the Americas, is a polyphagous pest with the greatest loss in maize [23,24]. Since 2019 when *S. frugiperda* first invaded China, it has invaded 27 provinces and attacked more than a dozen crops, mainly maize [25]. The pest area exceeds 1.3 million ha, and it has further spread to North and Northeast China [26]. *S. frugiperda* has become one of the highly destructive moth pests in China. Under noncontrolled conditions, the potential economic loss of maize caused by the invasive pest will be 60–500 billion dollars across China using the random model @RISK [27]. Therefore, the prevention and control of *S. frugiperda* are difficult. To protect the maize industry of China, large quantities of insecticides have been employed in the emergency chemical prevention and control of *S. frugiperda* to slow the spread of the pest and to minimize damage to maize since 2019 [28]. Emamectin benzoate (EB) is a semisynthetic insecticide derived from the avermectin family. In addition to its high efficiency, broad spectrum, and low toxicity, EB has better improved thermal stability, solubility, and insecticidal activity than avermectin. EB has been widely used for pest control, such as lepidopterous species, mites, and coleopterous and homopterous pests [29,30]. Currently, EB is recommended as an important emergency pesticide for the control of *S. frugiperda* based on the expert demonstration as there are no registered synthetic pesticides in China (accessed on 3 June 2019), and the extreme indoor contact toxicity and excellent control efficacy of EB against *S. frugiperda* also have been proved in maize production [31]. However, the current commercial formulations of emamectin benzoate are still mainly emulsifiable concentrates that comprise numerous highly toxic organic solvents [32,33]. Globally, scientists are shifting from sole dependence in overuse of chemical pesticides to integrated pest management (IPM) in order to protect the environment. Therefore, it is imperative to reduce the use of chemical pesticides in the control of *S.* *frugiperda* in China. In this study, EB was formulated as nanoparticles composed of DSPE-PEG_2000_-NH_2_ by the co-solvent method. The encapsulation of DSPE-PEG_2000_-NH_2_ can protect EB from adverse environmental conditions, such as high temperature and ultraviolet radiation, provide continuous release, as well as improve their foliar affinity by the functional groups on the nanoparticle surface. The formulation using DSPE-PEG_2000_-NH_2_ as a nanocarrier is biodegradable and environmentally friendly and especially eliminates the use of harmful solvents and adjuvants. The particle size, morphology, interfacial charge, contact angle (CA), retention, and bioavailability of the DSPE-EB nanoparticles were characterized to evaluate the formulation performance. The foliar affinity, insecticidal activity against *S.* *frugiperda,* anti-ultraviolet properties of the DSPE-EB nanoparticles, and a commercial pesticide were examined. This study provides an effective new method to significantly reduce the use of pesticides and to ensure the sustainable control of *S. frugiperda*. Furthermore, it contributes to IPM, green agriculture, and environmental protection.

## 2. Materials and Methods

### 2.1. Chemicals 

Emamectin benzoate (EB, 90% purity) and tetrahydrofuran (THF) were purchased from Energy Chemical Co., Ltd. (Shanghai, China), and 1,2-distearoyl-sn-glycero-3-phosphoethanolamine-N-[amino(polyethylene glycol)-2000] (DSPE-PEG_2000_-NH_2_) was obtained from Shanghai Yayi Biological Technology Co., Ltd. (Shanghai, China). Rhodamine 6G (R6G) was purchased from Shanghai Macklin Biochemical Co., Ltd. (Shanghai, China). Polyethylene 80 sorbitan monooleate (Tween 80) was purchased from Beijing Solarbio Technology Co., Ltd. (Beijing, China), and EB emulsifiable concentrate (0.57%) (EB-EC) was obtained from Shenzhen Noposion Agrochemicals Co., Ltd. (Shenzhen, China). 

### 2.2. Insect Collection and Rearing 

Larvae of the fall armyworm, *S.frugiperda,* were used to determine toxicity and were the laboratory strain used in this study. The original insects were collected from maize fields in Xintang village (26°21′ N, 119°05′ E), Minhou County in Fujian Province, China, in July 2021 and then reared on fresh maize leaves for one generation under the following conditions at 25 ± 1 °C with a photoperiod of 14:10 h (light: dark) and a 70 ± 5% relative humidity. 

### 2.3. Preparation of DSPE-EB Nanoparticles

The co-solvent approach, according to the literature, was employed to synthesize DSPE-EB nanoparticles [34]. First, we dissolved 5 mg of DSPE-PEG_2000_-NH_2_ and 0.1 g of EB in 1 mL THF under ultrasound treatment to form a homogeneous solution. Then, 9 mL of distilled water was added under ultrasound treatment. The above mixture was vigorously stirred overnight to remove THF and was filtered using a 0.22 μm syringe filter to obtain the final DSPE-EB nanoparticles. 

### 2.4. Characterization of DSPE-EB Nanoparticles

The particle size and zeta potential of the DSPE-EB nanoparticles were determined using a ZS-90 and Mastersizer 2000 (Malvern Instruments Ltd., Malvern, UK) in water through ultrasonic dispersion at a suitable concentration. The shape and surface morphology of the nanoparticles were observed under a transmission electron microscope (TEM, Hitachi HT7700, Hitachi Ltd., Tokyo, Japan). For TEM observation after the ultrasonic treatment of the nanoparticles, one drop of the suspension was deposited onto parafilm, and a carbon-coated copper grid was placed on top of the suspension drop for 5–10 min and then dried prior to observation.

### 2.5. Determination of Encapsulation Efficiency

The encapsulation efficiency (EE) of the DSPE-EB nanoparticles was calculated according to the literature [35]. The process was as follows: DSPE-EB nanoparticles amounting to 10 mL were centrifuged at 12,000 rpm for 20 min, and the supernatant was measured using an ultraviolet absorption spectrophotometer (TU-1810, Beijing Puxi General Instrument Co., Ltd., China) based on the standard curve equation A= 0.036C + 0.002 (R^2^ = 0.9999). The encapsulation efficiency (EE) was calculated by the following equation, where M_0_ is the initial amount of EB in the suspension before centrifugation, and M is the amount of EB in the nanoparticles.

$$\mathrm{EE}\%=\frac{M}{M_{0}}\times 100\%$$

### 2.6. Contact Angle Measurement

The contact angles of the DSPE-EB nanoparticles and EB-EC on maize leaves were measured, respectively, by a contact angle meter (FCA2000A, Shanghai Aifeisi Precision Instrument Co., Ltd., Shanghai, China) at room temperature; 5 µL of DSPE-EB nanoparticles and 5 µL of EB-EC were dropped onto the surfaces of fresh maize leaves, respectively. The droplets were photographed after 10 s. The contact angle was analyzed by the multipoint fitting method, and the average value of three replicates was calculated.

### 2.7. Maximum Retention Measurement

The retention measurement (Rm, mg/cm^2^) on leaves was measured according to the literature [36]. First, the DSPE-EB nanoparticles and EB-EC were diluted in 0.02% aqueous dispersions. Second, each leaf was weighed using an electronic analytical balance (Denver Instruments [Beijing] Co., Ltd., Beijing, China). Then, the leaves were fully immersed in the above dispersions and pure water, which was used as a control test. After 10 s, each leaf was removed from the solution and weighed again. The retention (R_m_) was calculated by the following equation, where W and W_0_ are the weights of the leaf before and after immersion in solution, and S is the leaf area. The average value of three tests was calculated.

$$R_{m}=\frac{W\;-\;W_{0}}{S}\;\times \;100\%$$

To visualize the performance of the DSPE-EB nanoparticle adhesion behavior on maize foliage, a fluorescent model drug, R6G, was selected. R6G was encapsulated within the DSPE-EB nanoparticles to obtain fluorescent-labeled DSPE-EB nanoparticles (R6G-DSPE-EB). Then, the live maize foliage was washed gently several times with deionized water to remove any dust on the surface. After natural drying in air, the R6G-DSPE-EB nanoparticles (400 µL, 4.0 mg/mL) were sprayed onto the surface of the maize foliage. After drying in air, the foliage was washed with deionized water (50 mL), dried, adhered smoothly onto glass slides, and then imaged by a confocal laser scanning microscope (CLSM) (Leica TCS SP8) at excitation wavelengths of 555 nm for R6G and 630 nm for chlorophyll. 

### 2.8. Sustained Release Measurement

The release profile measurements for EB from DSPE-EB nanoparticles were performed by a dialysis method [37]. The EB and DSPE-EB nanoparticles were placed into dialysis bags with a molecular weight cutoff at 3500 Da (Beijing Solarbio Science & Technology Co., Ltd., Beijing, China) and dialyzed against 45 mL of 40% (*v*/*v*) ethanol aqueous solution at 25 °C. At designated time intervals, 1 mL of the release medium was removed and replaced with an equivalent amount of 40% (*v*/*v*) ethanol aqueous solution. The concentrations of EB in the collected samples were analyzed by a UV–vis spectrometer. 

### 2.9. Insecticidal Activity Measurement

The insecticidal activities of the DSPE-EB nanoparticles and EB-EC were measured by the leaf dipping method under the laboratory conditions [38,39]. The insecticidal activity of DSPE-PEG_2000_-NH_2_ also was tested. The same concentration of DSPE-EB nanoparticles and EB-EC was diluted with deionized water to obtain samples of varying EB concentrations for testing the insecticidal toxicity. Based on a preliminary trial, serial concentrations of DSPE-EB nanoparticles and EB-EC ranging from 0.025 to 0.20 mg/L were tested with deionized water serving as a blank control. Clean and fresh maize leaves were cut into equal-sized pieces and were fully immersed in each DSPE-EB nanoparticle and EB-EC diluted solution for 10 s. After natural drying in air, each treated foliage piece was placed into a Φ = 6 cm cell culture dish and then five third-instar *S. frugiperda* larvae were released into the dish. The bioassay experiment of each concentration was replicated three times and was performed under the same conditions under which FAW were reared. The mortality of treated insects was observed and calculated 24 h, 48 h, and 72 h after the treatment.

The field efficacy trial was performed in Xintang Village (26°20′54″ N, 119°05′42″ E), Minhou County, Fujian Province, China. The maize variety used was xiantian 1. The trial object was *S. frugiperda* larvae. Three trials were created, namely, the test group (DSPE-EB nanoparticles), the reference group (EB-EC), and the blank group (water). The EB dose was set at 45 L final water solution/ha in different concentrations of EB (4 mg/L, 8 mg/L and 12 mg/L). These concentrations were sprayed using a knapsack sprayer. The decrease rate of the *S. frugiperda* larvae was evaluated after 1 d, 3 d, 7 d, and 14 d. Among the five sampling points randomly selected in each plot, five maize plants from each point were selected as the survey points. The experiment was repeated three times with different concentrations. The decrease rate was calculated using the following equation, where N_1_ and N_2_ are the number of insects before and after application, respectively, in the treatment area.

$$\mathrm{Decrease}\;\mathrm{rate}\;(\%)=\frac{N_{1}-N_{2}}{N_{1}}\;\times \;100\%$$

### 2.10. Statistical Analysis

The experimental data were analyzed using the SPSS Statistics 21. The effects of different concentrations of DSPE-EB nanoparticles and EB-EC on decrease rate of *S. frugiperda* were subjected to one-way analysis of variance (ANOVA) by Duncan’s multiple range test. The probability value *p* < 0.05 was considered statistically significant. The LC_50_ and LC_90_ used to evaluate the insecticidal activity were estimated by probit analysis.

## 3. Results 

### 3.1. Synthesis and Characterization of DSPE-EB Nanoparticles

**Scheme 1:**
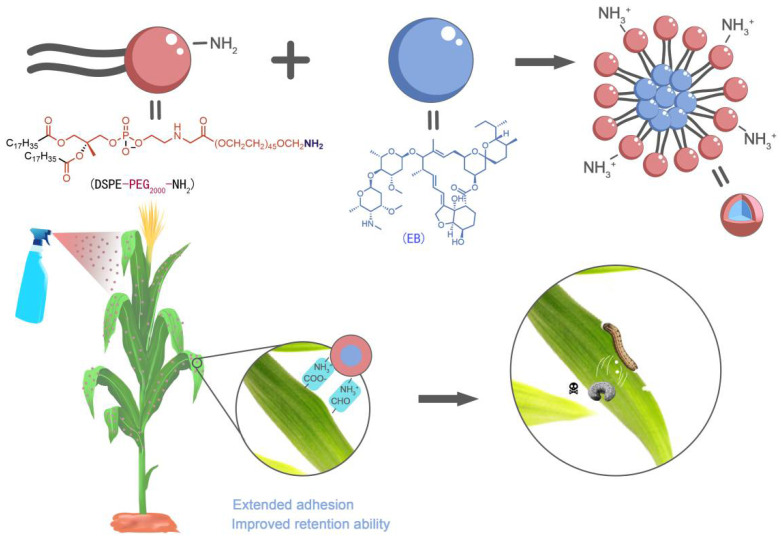
Schematic illustration of the preparation of DSPE-EB nanoparticles and their application for *S. frugiperda* control.

**Figure 1:**
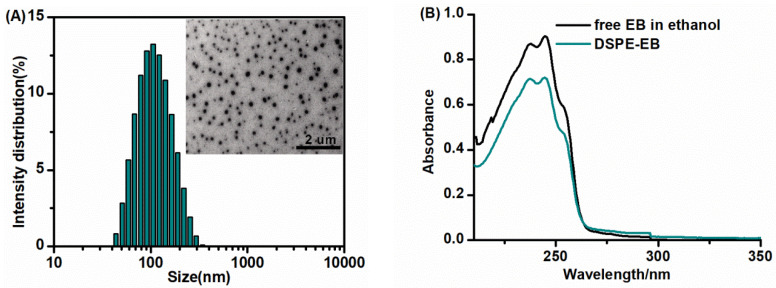
DLS size distribution and TEM images of DSPE-EB nanoparticles (**A**); the absorption spectra of DSPE-EB nanoparticles and free EB in ethanol (**B**).

The DSPE-EB nanoparticles were fabricated by a modified co-solvent method, as shown in Scheme 1. The synthesis procedure was simple and required only one reaction step. DSPE-PEG_2000_-NH_2_ was used to encapsulate EB to yield nanoparticles. The synthesis starts with the preparation of a THF solution containing EB (0.1 g) and DSPE-PEG_2000_-NH_2_ (5 mg). Upon mixing of the THF solution with water under continuous sonication, the hydrophobic lipid segments tended to be embedded in an aggregated hydrophobic EB core while the hydrophilic PEG chains extended into the aqueous phase to produce the nanoparticles. Dynamic light scattering (DLS) results reveal that the as-prepared DSPE-EB nanoparticles had a hydrodynamic diameter of 118 nm. The zeta potential of DSPE-EB nanoparticle is 17.7 mV. The transmission electron microscopy (TEM) image verified the formation of DSPE-EB nanoparticles with a well-defined spherical shape and homogeneous distribution (Figure 1A). In addition, the as-prepared DSPE-EB nanoparticles had good stability and dispersity in water due to no obvious change in their diameters for three months. The insecticide encapsulation efficiency (EE) of the nanoparticles is crucial for their field trial application. The EB encapsulation efficiency was 70.5 ± 1.5%, that is, the content of EB in the nanoparticles is 70.5 ± 1.5%.

The absorption spectra of the DSPE-EB nanoparticles were compared to free EB of the same concentration (Figure 1B). The peak absorbances of the DSPE-EB nanoparticles occurred at 237 nm and 245 nm, indicating that EB was successfully loaded into the DSPE-PEG_2000_-NH_2_ polymeric liposomes. 

### 3.2. Retention Property and Wettability of DSPE-EB Nanoparticles

#### 3.2.1. Wetting Performance Analysis

**Figure 2:**
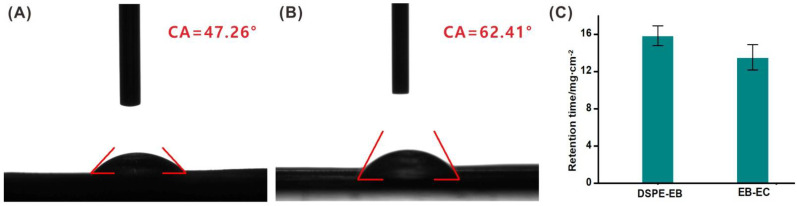
Contact angle of DSPE-EB nanoparticles (**A**) and EB-EC (**B**) on the surface of maize foliage; (**C**) the retention of the DSPE-EB nanoparticles and EB-EC solutions on maize leaves.

An important way to improve the effective utilization of pesticides is to improve their leaf spread performance on target crops. This study evaluated the contact angle of 0.02% aqueous solution of the DSPE-EB nanoparticles and EB-EC on the maize foliage. As shown in Figure 2A,B, the contact angles of the DSPE-EB nanoparticles and EB-EC were 47.26° ± 0.96° and 62.41° ± 1.37°, respectively. The smaller the contact angle of a droplet, the easier its distribution and spreading on the foliage surface of the targeted crops.

#### 3.2.2. Retention and Adhesion Ability Analysis

A good retention ability of pesticides can improve their bioavailability. The retention of the DSPE-EB nanoparticles and EB-EC solutions on the maize leaves were measured (refer to Section 2.6 for the experimental procedure). The retention of the DSPE-EB nanoparticles was measured to be 15.85 mg/cm^−2^ and was higher than that of EB-EC solution (13.52 mg/cm^−2^), as shown in Figure 2C. This may be because PEG is a long-chain polymer that helps to prevent droplet breakage and to reduce losses.

**Figure 3:**
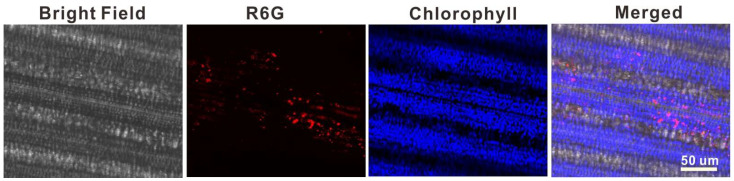
Confocal fluorescence images of DSPE-EB on the surface of maize foliage after washing with 10 mL deionized water (chlorophyll, λ_ex_: 630 nm, λ_em_: 670 nm, R6G, λ_ex_: 555 nm, and λ_em_: 580 nm).

R6G was chosen as a fluorescent dye for analysis of the adhesion behavior of the DSPE-EB nanoparticles on maize foliage by CLSM. As shown in Figure 3, after flushing with deionized water, the red fluorescent signal of R6G also was detected clearly in maize foliage, indicating a good affinity. This could be attributed to the amine groups on the surface of the DSPE-EB nanoparticles that interacted with the maize leaves through covalent bonding and electrostatic attraction [40].

### 3.3. Sustained Release

**Figure 4:**
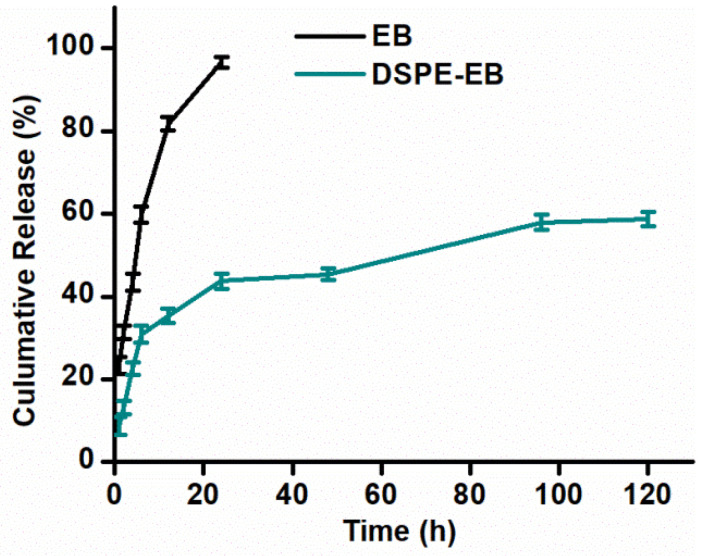
Sustained release curve of EB and DSPE-EB in 40% ethanol.

The sustained release properties of EB from the DSPE-EB nanoparticles and free EB were compared and are shown in Figure 4. In the 40% (*v*/*v*) ethanol aqueous solution, the release of EB was faster than that from the DSPE-EB nanoparticles. Within the initial 20 h, more than 90% of EB was released, while only 40% was released from the nanoparticles. Eventually, the release rates of EB from the DSPE-EB nanoparticles were gradual. The kinetic release profile of EB from the DSPE-EB nanoparticles can be described as a two-step process. During the initial stage, about 30% of EB was released within the first 6 h. Subsequently, the release rate slowed and the total release rate for 120 h was 58.8%, revealing a sustained release property. The initial rapid release was largely attributed to the pesticide existing at or near the surface of the nanoparticle. The slow-release process was due to pesticide entrapped in the DSPE-PEG_2000_-NH_2_ matrix [10]. 

### 3.4. Insecticidal Activity Analysis

**Figure 5:**
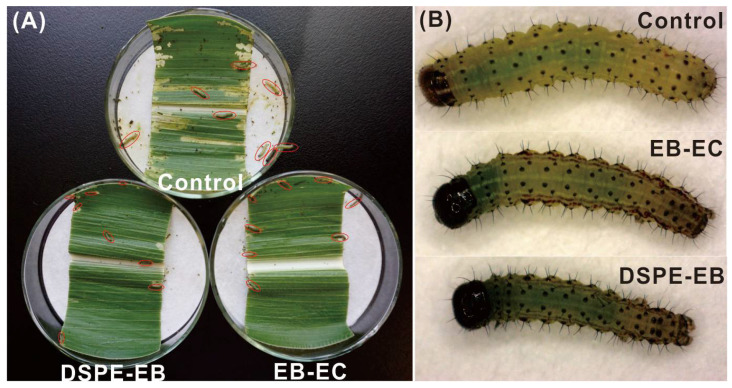
Image of maize leaves with 0.2 mg/L DSPE-EB nanoparticles and EB-EC against third-instar *S. furgiperda* larvae (**A**) after 24 h; the microscope magnified image of third-instar *S. furgiperda* larves treated with DSPE-EB nanoparticles and EB-EC after 24 h (**B**).

First, we tested the insecticidal activity of free carrier DSPE-PEG_2000_-NH_2_; it showed no toxicity toward *S. frugiperda* even at concentrations up to 1 mg/mL, indicating that it can be used as a suitable carrier for pesticide delivery. Then, the insecticidal efficiencies of the DSPE-EB nanoparticles and EB-EC were explored. As shown in Figure 5A, *S. frugiperda* was fed with the untreated maize leaves, and all insects were alive after 24 h. *S. frugiperda* was fed with the DSPE-EB nanoparticles and EB-EC, and the remaining blade area was larger than that in the control group, suggesting that the toxicity of EB loaded in the DSPE-EB nanoparticles was preserved. However, the insects that were fed 0.2 mg/L DSPE-EB nanoparticles and EB-EC treated leaves all died after the same time period. Figure 5B shows the length and growth of *S. frugiperda*, compared with the control sample, was slowed when fed the DSPE-EB nanoparticles and EB-EC treated leaves, and the tails of *S. frugiperda* were more likely to shrink and became black, showing signs of poisoning. 

**Figure 6:**
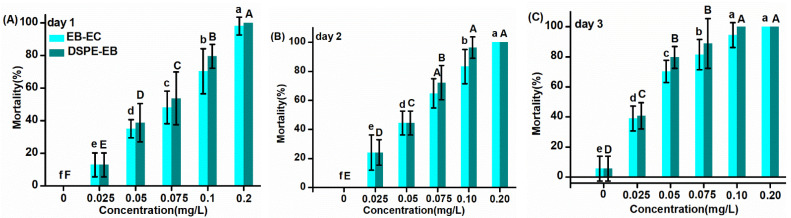
Mortality of different concentrations of EB-EC and DSPE-EB against third-instar *S. furgiperda* larvae at 1 (**A**), 2 (**B**) and 3 d (**C**) after treatment. Data with different lowercase letters (EB-EC) and uppercase letters (DSPE-EB) are significantly different at the *p* < 0.05 level according to Duncan’s multiple range test. In all the above data, the error bars represent the standard errors of the means of three replicates.

To further evaluate the biological activity of the DSPE-EB nanoparticles, the third-instar *S. furgiperda* larvae were selected as a model insect. Five concentrations were set, 0 mg/L, 0.025 mg/L, 0.05 mg/L, 0.075 mg/L, 0.1 mg/L, and 0.2 mg/L for DSPE-EB and EB-EC. The efficacy of DSPE-EB nanoparticles and EB-EC was evaluated and compared at d 1, 2, and 3. Figure 6 shows the derived biological activity results. Figure 6 shows the mortality rate caused by the DSPE-EB nanoparticles and EB-EC treatment at each concentration increased over time and was dose-dependent. Compared with EB-EC, the mortality rate of the third-instar *S. frugiperda* larvae caused by the DSPE-EB nanoparticles increased slightly, indicating that EB loading into the DSPE-EB nanoparticles did not change its physical and chemical properties. Table 1 lists the toxicity results of DSPE-EB using EB-EC as a control obtained by probit regression in SPSS. In detail, the LC_50_ values obtained from the corresponding toxicity regression equations for EB-EC and DSPE-EB were 0.051 and 0.046 mg/L, respectively, after 48-h treatment. The LC_50_ values in 24 h and 48 h do not differ significantly since the CL overlap. There was no significant difference between EB-EC and DSPE-EB nanoparticles on mortality of *S. frugiperda* larvae. 

**Figure 7:**
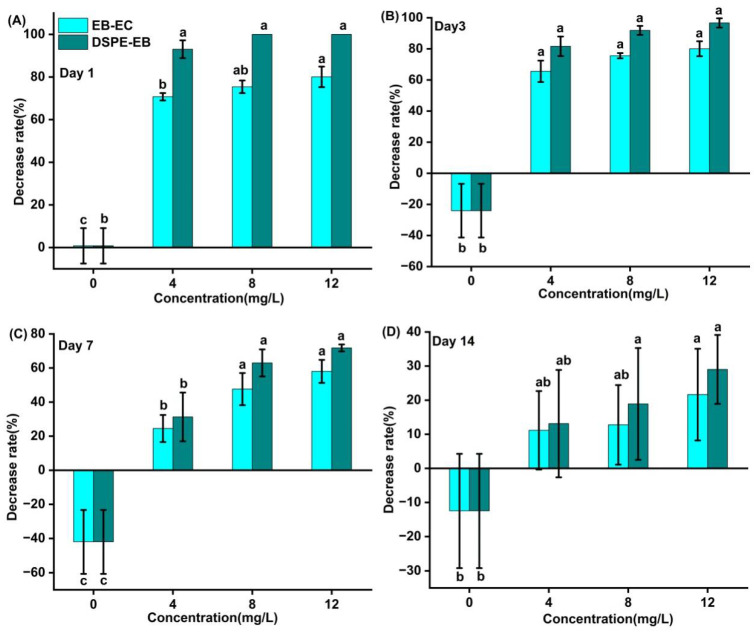
Decrease rates of different concentrations of EB-EC and DSPE-EB nanoparticles against *S. furgiperda* larvae at 1 (**A**), 3 (**B**), 7 (**C**), and 14 d (**D**) after spraying under field conditions. Different letters at each data indicate significant differences according to Duncan’s multiple range test at *p* < 0.05. In all the above data, the error bars represent the standard errors of the means of three replicates.

A field efficacy trial also was conducted. As shown in Figure 7, the DSPE-EB nanoparticles exhibited excellent insecticidal activity; the mortality rate of *S. frugiperda* at 4 mg/L was higher than 80% at 1 and 3 d after spraying. By contrast, the control group had a 20% increase in live *S. frugiperda* larvae. For DSPE-EB nanoparticles, the *S. frugiperda* death rate was slightly higher than that of EB-EC at different concentrations (4 mg/L, 8 mg/L and 12 mg/L), indicating strong control of *S. frugiperda*, which is probably because the DSPE-EB nanoparticles had strong leaf adhesion under complex outdoor conditions.

## 4. Discussion

In this study, we chose the amino-modified DSPE-PEG_2000_-NH_2_ to construct the nanoparticles, and its insecticidal activity was evaluated against an insect pest, *S. frugiperda*. In the foliar application of pesticides, the wetting and adhesion behaviors of pesticide on the foliage are of primary importance for affecting insecticidal activity [41]. An important way to improve the effective utilization of pesticides is to improve their wetting performance on target crops. Compared with the commercially available EB-EC, the contact angle of DSPE-EB nanoparticles on the surface of maize foliage was smaller, which indicated that DSPE-EB nanoparticles had better wetting ability on maize leaves. When EB was loaded into DSPE-EB nanoparticles, the specific surface area of the pesticide is increased. The small size, high adhesion ability, and retention, as well as the sustained release performance of the DSPE-EB nanoparticles could increase the contact probability between pesticide and target organisms. The wax layer on the maize foliage consists of many kinds of higher fatty acids and higher fatty aldehydes. These polar groups on the maize foliage surface may interact with the –NH_2_ groups on the DSPE-EB nanoparticles surface through covalent bonding and electrostatic attraction. The surface of the maize leaf is mainly composed of a hydrophobic waxy layer, which contains a large amount of fats and fatty acids. The hydrophilic DSPE-EB nanoparticles surface contains a lot of polar –NH_2_ groups, which are easily deposited at the leaf-fluid surface [36]. In addition, PEG is a long-chain polymer that helps to prevent droplet breakage and to reduce losses. Due to the high cost and low resolution of radioisotope technology, it is difficult to realize visualization, while fluorescence imaging has advantages of high sensitivity, convenience, and live sample scanning, which is used to investigate the deposition and adhesion behavior of nanoparticles on maize foliage. R6G was encapsulated within the nanoparticles, which allows visualization of the distribution of nanoparticles.

The leaf-dipping method was used to determinate the indoor toxicity of DSPE-EB nanoparticles and EB-EC. Compared with EB-EC, the DSPE-EB nanoparticles showed an improved ability to control *S. frugiperda*, especially under the field condition. Shen et al. report multifunctional nanoplatform, carboxymethyl chitosan-modified carbon nanoparticles as the carrier for EB via simple physisorption process; the nanoformulation showed improved solubility and dispersion stability in aqueous solution, exhibited pH-responsive controlled release performance, and enhanced anti-UV property, leading to superior pest control performance [42]. The insecticidal activity differences between EB-EC and DSPE-EB nanoparticles can be explained as active ingredient in the nanoparticles provided an effective way to avoid the photolysis and prolong the effective duration of EB [16]. Meanwhile, high adhesion ability and retention, as well as the sustained release performance of the DSPE-EB nanoparticles, lead to a high insecticidal performance. However, to achieve a sustainable agriculture, further studies are required to determine the effects of nanoparticles on natural enemies and the environment.

## 5. Conclusions

In summary, a new type of EB nanoparticle was prepared by the co-solvent method. Morphology and size, encapsulation efficiency, wetting and retention performance, sustained release performance, and insecticidal activity were investigated. As observed by TEM, the nanoparticles were spherical. The nanoparticles had smaller contact angles than EB-EC. The retention of the nanoparticles on maize leaves was nearly 1.4 times higher than that of EB-EC. In addition, R6G was selected to visualize the adhesion behavior of DSPE-EB nanoparticles in maize leaves by CLSM. DSPE-EB nanoparticles had strong adhesion on maize foliage, which depends on the amine groups on the nanoparticle surface. In addition, the nanoparticles exhibited sustained release behavior. In view of the simple preparation, excellent anti-pest activity, and lack of toxic organic solvent and adjuvants, these environmentally friendly nanoparticles have prospects for pest control and reducing pollution in the environment.

## Figures and Tables

**Table 1:** Indoor toxicity results of DSPE-EB nanoparticles and EB-EC against the third-instar *S. furgiperda* larvae.

Sample	Time	Toxicity Regression Equation	LC_50_ (mg/L)	LC_90_ (mg/L)	95% Confidence Limit
DSPE-EB	24 h	y = 4.307 + 3.518×	0.060	0.138	0.053–0.067
	48 h	y = 4.822 + 3.641×	0.046	0.103	0.040–0.051
EB-EC	24 h	y = 3.926 + 3.309×	0.065	0.159	0.057–0.074
	48 h	y = 4.187 + 3.243×	0.051	0.127	0.044–0.058

## Data Availability

We will provide all data generated in this study upon request.
